# Morphodynamic signatures of MDA-MB-231 single cells and cell doublets undergoing invasion in confined microenvironments

**DOI:** 10.1038/s41598-021-85640-5

**Published:** 2021-03-22

**Authors:** Xingjian Zhang, Trevor Chan, Michael Mak

**Affiliations:** Department of Biomedical Engineering, Yale University, New Haven, CT USA

**Keywords:** Biological fluorescence, Cancer imaging, Metastasis

## Abstract

Cancer cell metastasis is a major factor in cancer-related mortality. During the process of metastasis, cancer cells exhibit migratory phenotypes and invade through pores in the dense extracellular matrix. However, the characterization of morphological and subcellular features of cells in similar migratory phenotypes and the effects of geometric confinement on cell morphodynamics are not well understood. Here, we investigate the phenotypes of highly aggressive MDA-MB-231 cells in single cell and cell doublet (an initial and simplified collective state) forms in confined microenvironments. We group phenotypically similar single cells and cell doublets and characterize related morphological and subcellular features. We further detect two distinct migratory phenotypes, fluctuating and non-fluctuating, within the fast migrating single cell group. In addition, we demonstrate an increase in the number of protrusions formed at the leading edge of cells after invasion through geometric confinement. Finally, we track the short and long term effects of varied degrees of confinement on protrusion formation. Overall, our findings elucidate the underlying morphological and subcellular features associated with different single cell and cell doublet phenotypes and the impact of invasion through confined geometry on cell behavior.

## Introduction

The tumor microenvironment comprises a heterogeneous cancer cell population^1–5^. Recent studies have found subsets of tumor cells that exhibit a more invasive phenotype and a greater potential to initiate metastasis^6–9^. The process of cancer metastasis is understood as a series of cell transport events, beginning with cells invading through physical constraints of the primary site, followed by transendothelial migration, and culminating in the colonization of a secondary tissue^1,8^.

At various stages of metastasis, cells must overcome mechanically constrictive barriers to migration, such as small pores in the extracellular matrix (ECM) of dense stromal tissue. Migratory phenotypes and cell morphologies are linked. A recent study has shown that the metastatic potential of cancer cells in vivo may be related to the morphologies of those cells on a 2D substrate^10^. This study supports 2D cell morphology as a possible indicator of cancer cell metastatic potential. Changes in cell morphodynamics and migratory phenotype are ultimately the result of subcellular activities. For example, morphological changes can arise from cytoskeletal remodeling through actin polymerization and myosin-mediated contractions^11–13^, and persistent and fast cell movement is found to be associated with anterior localization of mitochondria (relative to the location of the nucleus) in both unconstrained and constrained environments^14^.

Cancer metastasis can involve a single cell or a small tumor cluster interacting and migrating collectively^15–18^. Many factors affect the number of cells that escape from the primary tumor site. In some instances, multiple cancer cells migrate together in a leader–follower manner^15, 16^. Recent studies also suggest that leader cells and follower cells can switch dynamically based on their energetics^17,18^. Furthermore, cancer metastasis can occur over a prolonged period during which cells can divide. Small tumor clusters have also been found in circulating tumor cells^16^. Thus, the analysis of cell doublets may serve as an important simplified type of collective migration beneficial to understanding the migratory behaviors of small tumor clusters.

We previously developed a microfluidic multi-staged serial invasion channels (MUSIC) device platform with parallelized constriction channels in series that mimic the constrictive barriers in the microenvironment. This device has been used to measure cancer cell deformation and relaxation, drug treatment’s impact on cancer cell invasiveness, etc^19–21^. In addition, we previously developed a custom fluorescence analysis approach to determine distinctions in cell morphology, cytoskeletal activity, and mitochondrial activity under various geometric constraints^22^.

Here, we apply the MUSIC device, together with custom fluorescence analysis methods, to identify the morphological and subcellular features associated with different migratory phenotypes of MDA-MB-231 metastatic breast cancer cells. We further examine the impact of different levels of confinement on short and long term morphological states.

## Results

### Heterogeneous single cell migratory phenotypes

**Figure 1:**
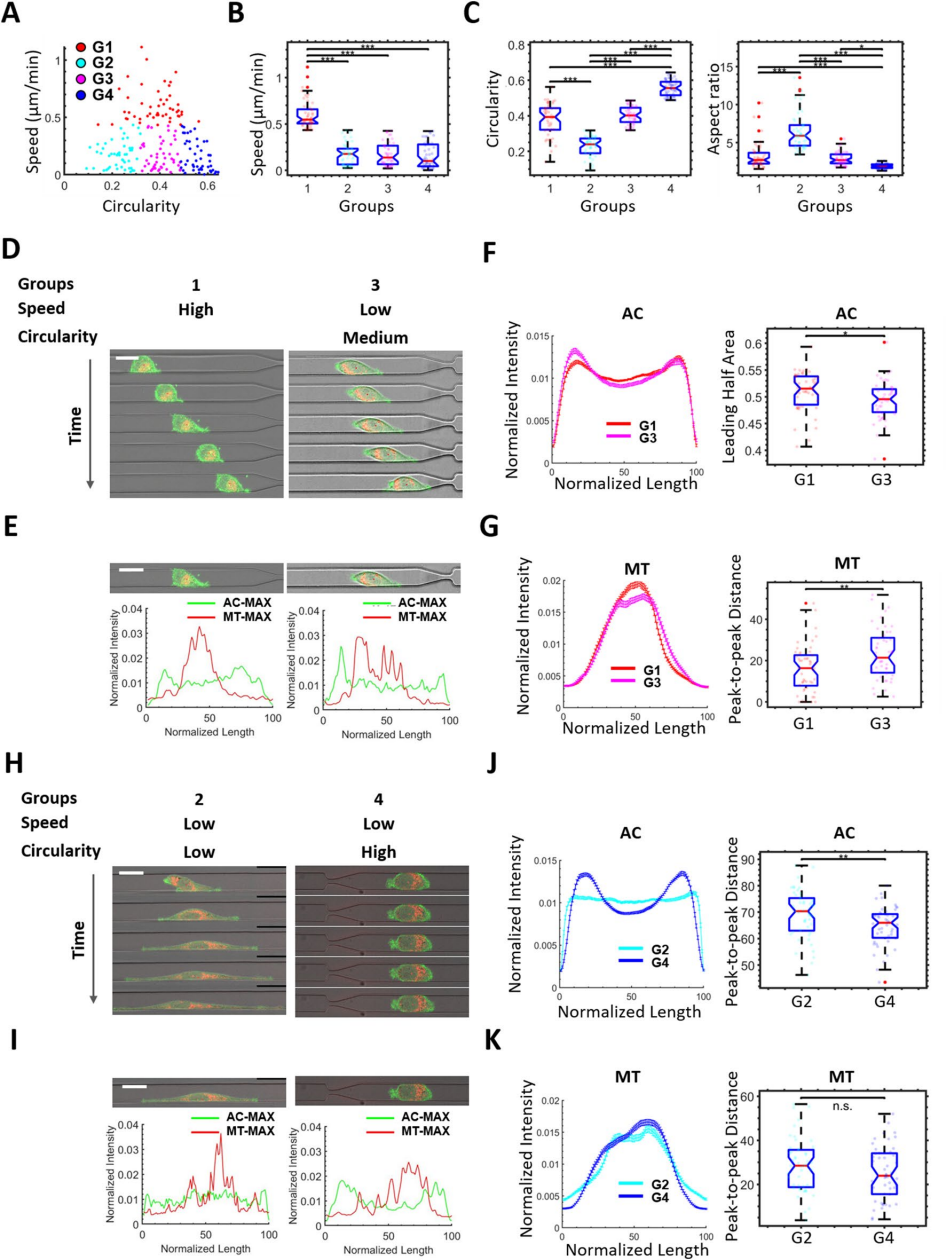
The behavior of single cells. (**A**) The speed vs. circularity relationship of all cells (N = 195) in the wide channel is shown in the scatter plot. The cells are separated into 4 similar groups, G1-G4. (**B**) Speed is used to compare the cells across the groups. (**C**) Circularity (left) and aspect ratio (right) are used to compare the cells across the groups. (**D**, **H**) The comparison of representative cell time-lapse images between G1 (**D**, left) and G3 (**D**, right), and between G2 (**H**, left) and G4 (**H**, right). Frame interval is 40 min, scale bar is 20 μm. (**E**, **I**) The comparison of representative cell images between G1 (**E**, left) and G3 (**E**, right), and between G2 (**E**, left) and G4 (**E**, right), the corresponding normalized 1D actin and mitochondria profiles are shown below. Scale bar is 20 μm. (**F**, **G**) The average actin (**F**) and mitochondria (**G**) profiles are used to compare between G1 (N = 49) and G3 (N = 49). (**J**, **K**) The average actin (**J**) and mitochondria (**K**) profiles are used to compare between G2 (N = 49) and G4 (N = 48). The standard error of the mean is used as the error bars in the profile plots. The box plots on the right side are statistical comparisons of selected variables used to extract differences between the compared profiles.

Within the wide channel of our device, which mimics less constrictive gaps in the ECM, we find cells exhibit a wide range of circularities and speeds, indicating phenotypic heterogeneity in the population (Fig. 1A). We categorize phenotypically similar cells (based on cell speed and/or circularity, see Methods ‘Variables definition’ for details) into four similar-sized groups (G1-G4). Grouping cells with similar morphologies and migratory behaviors allows us to extract associated subcellular features. Specifically, G1 encompasses the highest 25% of the cells in speed (G1: speed > 0.434 μm/min); the remaining 75% are separated into 3 equal groups based on circularity (G2: low, circularity < 0.318, G3: middle, 0.318 < circularity < 0.487, G4: High, circularity > 0.487), resulting in each group containing 25% of the total population (Fig. 1B,C).

To process the fluorescence signal, we perform maximum intensity z and y projections of the 3D actin (AC) and mitochondria (MT) fluorescence signal. We then normalize the profile to a fixed length along the x-dimension (the long axis of the channel in which the cell migrates) and area so that relative fluorescence intensity signals can be compared. We further flip the fluorescence profile based on the direction of cell migration so that the right side of the profile (x = 100) always corresponds to the leading edge of the cell (see Methods ‘Variables definition’ for details).

To understand the association between subcellular activities and cell migratory phenotypes, we compare the two groups (G1 and G3) with similar circularity and aspect ratio but different cell speed (Figs. 1B,C). Although the cells from the two groups appear similar under the microscope (Figs. 1D,E), the profile analysis indicates a significantly larger actin profile leading half area (Fig. 1F, area under the leading half of the profile, see Methods ‘Variables definition’ for variable details), and a lower mitochondria profile peak-to-peak distance (Fig. 1G, distance between the two peaks, see Methods ‘Variables definition’ for details) in G1 compared to G3. This indicates that cells in the fast group have a greater actin fluorescence intensity in their leading half and more centralized mitochondria.

We further compare the two groups (G2 and G4) with similar speeds (Fig. 1B) but with significantly different cell circularities and aspect ratios (Figs. 1C,D). The morphologies of cells in G2 are elongated with protrusions formed at both sides of the cells, whereas cells in G4 are not elongated. The actin profile of G2 shows a more uniform intensity distribution across the cell with a significantly higher profile peak-to-peak distance (Fig. 1J). No obvious difference is detected in the mitochondria profile (Fig. 1K). These results suggest that elongated cells require a uniform actin intensity throughout the cell body, and that cell elongation is not necessarily associated with effective cell migration.

### Distinct migratory phenotypes in the fastest group

**Figure 2:**
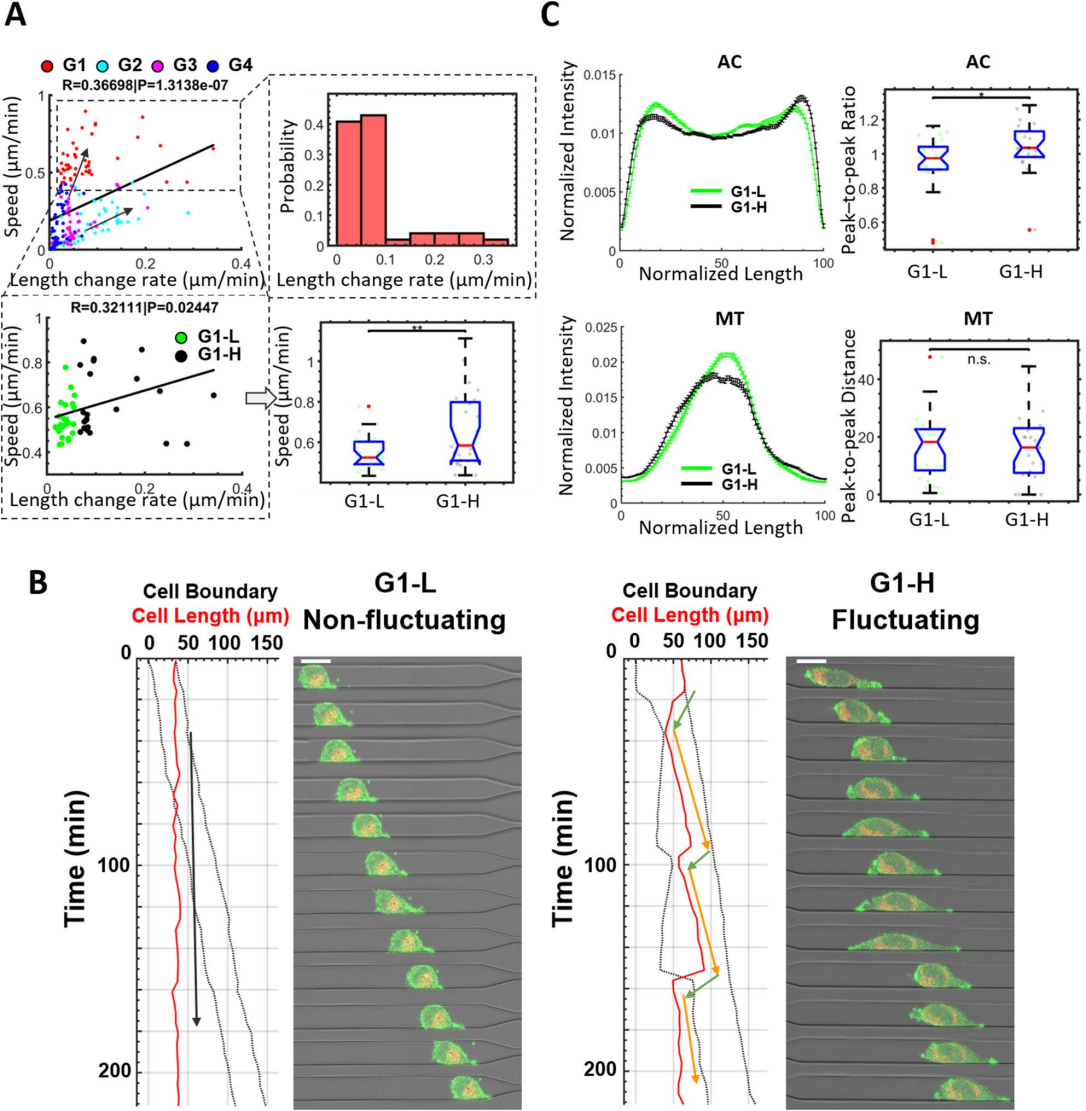
Two distinct migratory phenotypes in the fastest group G1. (**A**) Upper left: The speed vs. length change rate relationship (absolute value of the rate of cell length change) for all cells in the wide channel is shown in the scatter plot. The solid black line is the linear fit of the scatter plot. R and P values are shown on the title. R is Pearson’s correlation coefficient. The cells are color-coded based on the group. The black arrows point along with the two phenotypes, fluctuating (high speed, high length change rate) and non-fluctuating (high speed, low length change rate). Upper right: The probability plot shows the distribution of cell length change rate in the fastest group G1. Lower left: The speed vs. length change rate of the cells in G1. The cells in G1 are separated into two equal-sized subgroups, G1-L (Low length speed, N = 25) and G1-H (High length speed, N = 24). Lower right: The speed of the cells is shown to compare the two subgroups. (**B**) The time-lapse images of representative cells from each subgroup (Left: non-fluctuating; Right: fluctuating) show the dynamics of cell morphology during migration, with the corresponding quantification of cell boundary and cell length displayed on the left. The dotted curves indicate the leading and trailing edge of the cell over time. The red curves indicate cell length dynamics. The arrows guide visualization of the elongation-contraction cycles. Green arrows indicate cell contraction and orange arrows indicate cell elongation. Frame interval is 20 min, scale bar = 20 μm. (**C**) The average actin (upper-left) and mitochondria (lower left) profiles are used to compare between G1-L and G1-H. The standard error of the mean is used as the error bars in the profile plots. The box plots on the right side are statistical comparisons of selected variables used to extract differences between compared profiles.

Cell speed and the rate of change in cell length (taken as an absolute value of the rate of change in cell length) are positively correlated (Fig. 2A, upper-left). When we take a closer look at the cells in G1, a wide distribution of length change rate (Fig. 2A upper right) is detected, suggesting that cells within the fast migration group may exhibit distinct migratory phenotypes. To characterize the actin and mitochondria features associated with the difference in cell length dynamics within the fast migrating cells, we divide G1 into two equal-sized subgroups based on their length change rate G1-L (low length change rate, i.e. non-fluctuating) and G1-H (high length change rate, i.e. fluctuating) (Fig. 2A, lower left). A significantly higher speed is detected in G1-H (Fig. 2A, lower-right). Morphologically, the cells in G1-L migrate with a relatively static geometry (Fig. 2B, left), whereas the cells in G1-H subgroup migrate via elongation-contraction cycles (Fig. 2B, right). In addition, we also detect a significantly higher actin profile peak-to-peak ratio (Fig. 2C upper, the ratio of leading peak intensity/trailing peak intensity, see Methods ‘Variables definition’ for details) in the actin profile of G1-H, indicating stronger actin activity at the leading side is associated with cell fluctuation and faster cell migration. No obvious difference is detected in the mitochondria profiles (Fig. 2C lower).

### Heterogeneous cell doublet migratory phenotypes

**Figure 3:**
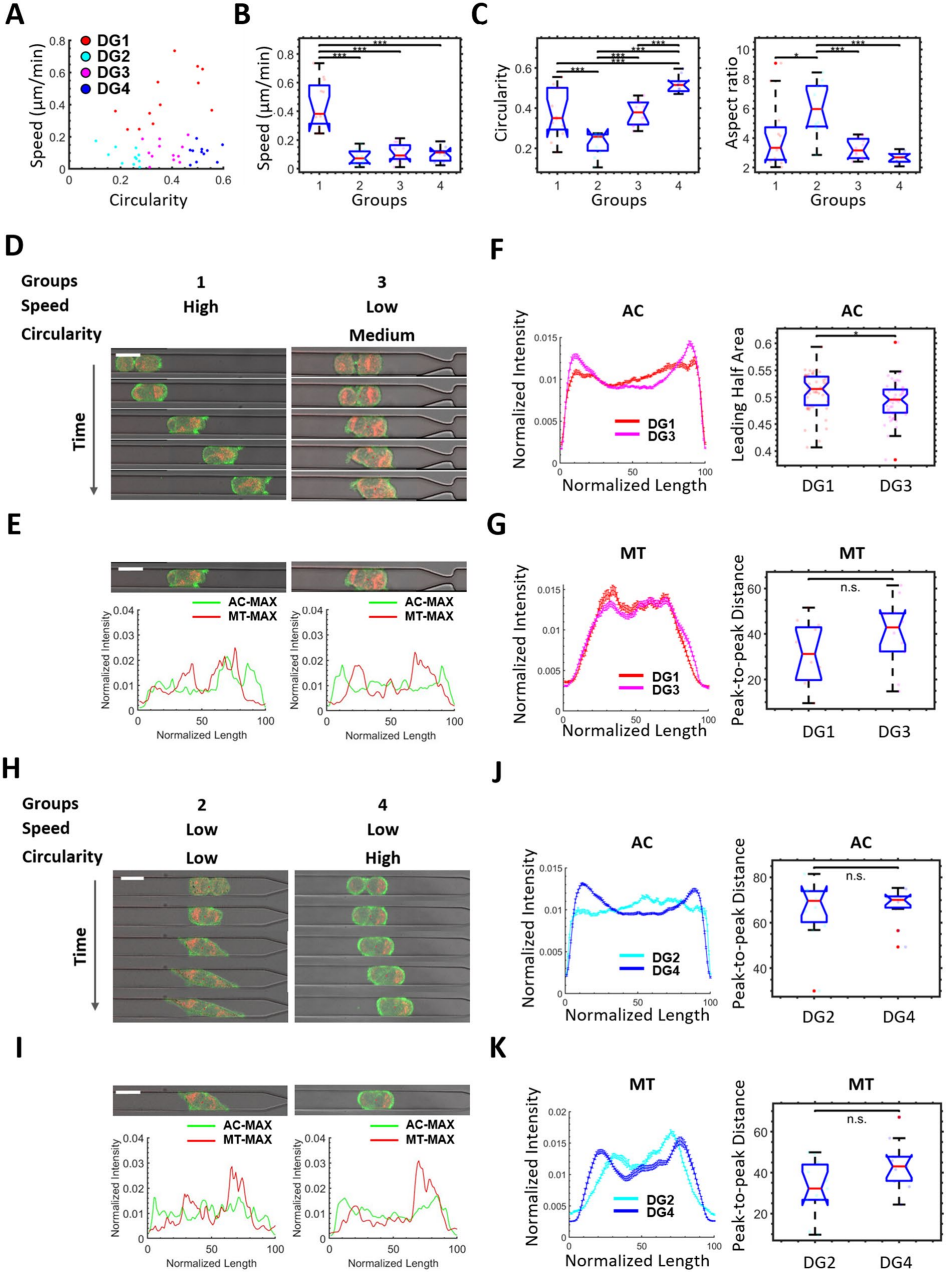
The behavior of cell doublets. (**A**) The speed vs. circularity relationship of all cell doublets (N = 46) in the wide channel is shown in the scatter plot. The cells are separated into 4 similar groups, DG1-DG4. (**B**) Speed is used to compare the cells across the groups. (**C**) Circularity (left) and aspect ratio (right) are used to compare the cells across the groups. (**D**, **H**) The comparison of representative cell time-lapse images between DG1 (**D**, left) and DG3 (**D**, right), and between DG2 (**H**, left) and DG4 (**H**, right). Frame interval is 40 min, scale bar is 20 μm. (**E**, **I**) The comparison of representative cell images between DG1 (**E**, left) and DG3 (**E**, right), and between DG2 (**E**, left) and DG4 (**E**, right), the corresponding normalized 1D actin and mitochondria profiles are shown below. Scale bar is 20 μm. (**F**, **G**) The average actin (**F**) and mitochondria (**G**) profiles are used to compare between DG1 (N = 12) and DG3 (N = 12). (**J**, **K**) The average actin (**J**) and mitochondria (**K**) profiles are used to compare between DG2 (N = 11) and DG4 (N = 11). The standard error of the mean is used as the error bars in the profile plots. The box plots on the right side are statistical comparisons of selected variables used to extract differences between the compared profiles.

As cell invasion in vivo often occurs in a collective manner, where cell–cell interactions play important roles, we assess the behavior of cell doublets. We analyze the cell doublets that do not separate from each other after the initial cell division event. We apply the grouping and analytical methods used for the single cells (Fig. 1) to the cell doublets (Figs. 3A–C).

The comparison between cell doublet DG1 and DG3 (Figs. 3D,E) shows a significantly higher average actin profile leading half area (Fig. 3F) in DG1, with no significant difference detected between the mitochondria profiles (Fig. 3G). The comparison between cell doublet DG2 and DG4 (Figs. 3H,I) shows a more uniform actin profile in DG2 (Fig. 3J). No significant difference is detected in the actin and mitochondria profile peak-to-peak distance (Figs. 3J,K). When we compare the morphology of the cell doublets across the 4 groups, we find cell doublets in the slow group tend to form protrusions in opposing directions, or do not form protrusions, whereas doublets in the fast group form protrusion(s) in one direction (Figs. 3D,H). Overall, in terms of cell doublet polarization and migration, we find that cell doublet groups exhibit similar morphological features and subcellular actin and mitochondrial distributions as their corresponding single cell groups, indicating that factors driving polarization and migratory phenotypes in collective migration may mirror those responsible in single cell migration.

### Impact of confinements on cancer cell protrusion formation

**Figure 4:**
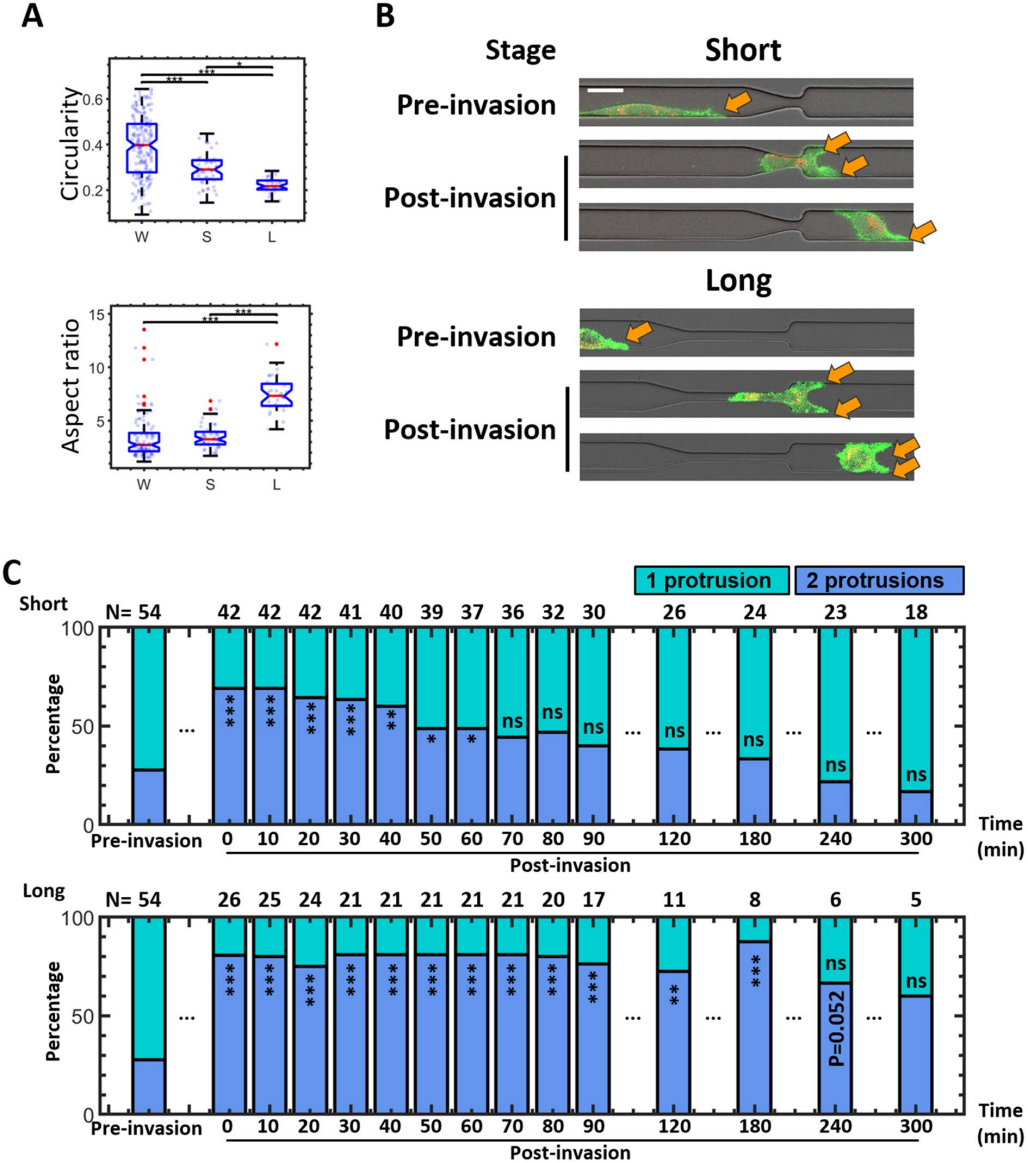
Impact of geometric confinement on cancer cell protrusion formation. (**A**) Circularity (upper) and aspect ratio (lower) are used to compare cells in the wide (W, N = 195), short constriction channel (S, N = 50), and long constriction channel (L, N = 32). (**B**) The representative cell images show cell protrusion formation at the leading edge when migrating through a short (left) or a long (right) constriction channel from pre-invasion to post-invasion stage. The arrows indicate the protrusions. Scale bar = 20 μm. (**C**) The composition of cells with 1 or 2 protrusions over time is used to show protrusion dynamics. Cells are categorized based on their leading edge protrusion number at each time point. The upper is cells that migrate through the short constriction channels, and the lower is cells that migrate through the long constriction channel. The legends show the category of cell protrusion number. The N number above each bar is the sample number used at each time point. The y-axis is the percentage of the cells with corresponding leading edge protrusion numbers. The composition of protrusion at each time point of post-invasion is compared with pre-invasion separately.

We then detect the impact of geometric confinements on cancer cell morphology. Our device provides two types of constriction channels to mimic different levels of confinement in the physiological system. Both short and long constriction channels (Fig. 4B, upper) have a minimum cross-sectional profile of 7.0 μm (width) by 6.8 μm (height). The short constriction channel, designed to mimic various ECM pores (e.g. in fibrillar collagen matrices), has a length of 10 μm. The long constriction channel has a length of 60 μm and is designed to mimic more confined spaces such as dense ECMs and microcapillaries, both of which require cells to undergo large deformations in order to migrate through. We quantify the deformation induced by the geometry by measuring cell circularity and aspect ratio. A significant reduction of cell circularity is detected in the short and long constriction channels. A significantly higher cell aspect ratio is further detected in the long constriction channel (Fig. 4A).

To test the short term and long term impact of the deformation on cell morphological phenotype, we defined two stages of cell migration: pre-invasion and post-invasion. Pre-invasion is defined as 1 h before the leading edge of a cell reaches the entrance of either a short or the long constriction channel. This stage is used to determine the cell protrusion phenotype prior to geometric compression. The protrusion composition of cells in the pre-invasion stage distributes similarly in comparison to that of the motile subset of cells that stay inside the wide channel (Supplementary Fig. 1). Post-invasion begins when the center of mass of the cell (defined based on 2d binarized cell image) exits the constriction channel (t = 0 min) and continues until the cell stops migrating, or until we lose track of the cell due to imaging duration and field of view limitations. This resulted in a decrease in N number over time.

Our results show that the incidence of cells exhibiting two protrusions at the leading edge increases instantly after the cells migrate through the constriction channels (Fig. 4B,C, post-invasion t = 0 min). A time-dependent memory effect on the formation of two leading protrusions is shown in both short and long constriction conditions (Fig. 4C, post-invasion, t > 0). We detect that the two protrusion formation induced by short constriction channels can last around 60 min (Fig. 4C, upper), whereas long constrictions, which induce larger strains on the cells, produce a longer lasting two protrusion phenotype (Fig. 4C, lower).

## Discussion

Tumor heterogeneity complicates disease treatment by enhancing drug resistance^23^ and metastasis^1^. Identifying specific morphological features and their associated migratory phenotypes could facilitate the determination of invasive subpopulations through analyzing static images^10^. Using a microfluidic system with confined geometries and custom image analysis, we group MDA-MB-231 cells by morphological features and migratory behavior and compare the resulting subcellular profiles.

Based on the results, we summarize the factors relevant for a single cell to elongate and migrate. To facilitate cell elongation, cells form protrusions at both the leading and trailing edges and exhibit comparatively uniform actin intensity (Fig. 1H, J). For increased migratory phenotypes, cells form an asymmetrical protrusion geometry with major protrusion(s) formed at the leading edge of the cells (Fig. 1D,E). These findings show results consistent with previous studies about the function of protrusive structures in cancer cell invasion^12,24^. In addition, actin and myosin have been shown to play important roles in cancer cell migration^11,13^. In this study, we identify multiple subcellular features including intensified actin at the leading half of the cell (Fig. 1F) and centralized mitochondria (Fig. 1G), which are correlated with fast and persistent movement.

Within the fast migrating group G1, we also observe two modes of migration: fluctuating and non-fluctuating (Fig. 2B). Cell elongation is usually accomplished through the formation of protrusive structures induced by underlying actin polymerization^11,25^, whereas cell contraction is shown to be mediated by activated myosin^25^. Close synchronization of myosin-mediated contraction and actin polymerization-mediated protrusion may result in static cell shapes, whereas asynchronous protrusion and contraction activities may result in strongly fluctuating cell shapes. In this study, we find the fluctuating group maintains a faster cell speed (Fig. 2A, lower right) with higher actin intensification at the leading side. This indicates that asynchronous protrusion and contraction promote a migratory phenotype for MDA-MB-231 cells in confined microfluidic channels. Follow up studies exploring the underlying mechanisms that lead to synchronous and asynchronous protrusion and contraction phenotypes could further benefit our understanding of cancer migration.

Cancer cell metastasis often involves multiple cells migrating collectively^15–18^. Cancer cell division is a common event that occurs during metastasis. In the case of adherent cell doublets, migration is no longer determined by subcellular activity in a single cell, but by a continuous and dynamic mechanical interaction between the two daughter cells. In this study, we analyze the morphological and subcellular features of cohesive MDA-MB-231 cell doublets. Our results show that the same morphological features and subcellular actin and mitochondrial profiles that correlate to single cell speed and specific morphological phenotypes also correlate to cohesive cell doublet speed and morphology. Potential future studies could examine whether the associations between heterogeneous morphodynamics and cell migratory phenotypes observed in this study are applicable to other tumor cell types, e.g. epithelial vs mesenchymal and invasive vs non-invasive.

Finally, during invasion, cells are squeezed by the surrounding geometry as they pass through constricted microenvironments. Examining the effect of geometric confinements on cancer cells and the temporal evolution of cell behavior before and after invasion can provide insights toward how cells are altered after passing through constrictions. Our results indicate that after MDA-MB-231 cells migrate through the constriction geometries, the percentage of cells exhibiting two leading protrusions increases. Previous studies have shown that cells can form focal adhesions at protrusions to generate mechanical forces^24,26–28^. The increase in protrusion formation detected in this study could potentially be associated with increase in mechanical forces generated by cells. Further studies involving visualizing the formation of focal adhesion sites on the protrusions can provide more insights toward the mechanical role of increased protrusion formation on cancer cell invasion. Based on tracking the two protrusion phenotype after invasion, our results show a difference in the duration of this state between cells that migrate through short constriction channels and cells that migrate through long constriction channels. The protrusion increase can last up to 240 min after the cell exits the long constriction channel, far longer than the 60 min duration observed after cells exit the short constriction channel. According to previous studies, the cytoskeleton can form directional memory due to external stimuli, such as chemical gradients^29^. In our study, we observe a memory effect in cell protrusion formation as a result of mechanical stimuli due to geometric confinement. Furthermore, the degree of confinement may impact the duration of this memory effect, with large strains induced by highly constrictive barriers causing a much more durable memory effect in cytoskeletal and protrusion activity.

## Conclusion

Our study elucidates the phenotypic diversity of cancer cells during migration and invasion. There are multiple morphological signatures associated with fast moving cells, marked by distinct morphodynamics. Furthermore, as cells divide during invasion, cohesive doublets are formed, a potential means of initializing collective invasive clusters. Cell doublets behave as a unit that share similar attributes to individual cells. Finally, as cells undergo invasion through highly constrictive geometries, memory effects on cell morphology are generated, the duration of which are dependent on the degree of confinement. These findings highlight intrinsic and inducible features in cancer migration phenotypes.

## Methods

### Device fabrication and channel geometry

The device’s mask was designed in LayoutEditor and fabricated in the Yale School of Engineering and Applied Science cleanroom. Device fabrication follows the standard soft-lithography method mentioned in previous research^19, 30^. Briefly, SU-8 (SU8-2015, Newton) was spin-coated on a silicon wafer and exposed under the photomask to create a negative pattern. Then, mixed Polydimethylsiloxane (PDMS) (Sylgard 184, Dow Corning) with a weight ratio of 10:1 was poured onto the master. The PDMS mold was then peeled from the wafer and bonded to a glass coverslip. Another PDMS layer with a large reservoir connecting the two wells was bonded on top of the device, and a thin layer of PDMS lid was prepared to cover the large reservoir after cell loading.

The geometry of the channels are (1) Wide channel (W): Width: 19.0 μm, Height: 6.8 μm (w*h = 129.2μm^2^), 20 μm away from the boundary of the constriction channel; (2) Short Constriction (S): Width: 7.0 μm; Height: 6.8 μm (w*h = 47.6μm^2^); Length: 30 μm in total, with 10 μm for the constriction channel and 10 μm expansion on both sides of the constriction channel; (3) Long Constriction (L): Width:7.0 μm; Height: 6.8 μm (w*h = 47.6μm^2^); Length: 80 μm in total, with 60 μm for the constriction channel and 10 μm expansion on both sides of the constriction channel.

### Cell culture and mitochondria staining

MDA-MB-231 cells were transfected with LifeAct-GFP to display actin fluorescence. These cells were a kind gift from the Lauffenburger Lab at MIT. Dulbecco’s Modified Eagle Medium (DMEM) (Life Technologies) with 10% fetal bovine serum (Catalog#16000044, Gibco) and 1% penicillin–streptomycin (Life Technologies) are used as culture media. The cells are cultured in an incubator at 37 °C, 5% CO_2_. Cells were stained with 500 nM Mitotracker Deep Red Solution (M22426, Invitrogen) for 90 min then washed by DMEM before cell loading. Studies have shown that cell proliferation inhibitors, such as mitomycinC and Paclitaxel, can lead to changes to cell size^31^ and migratory phenotypes^21,32^. Therefore, to maintain physiological relevance, the cells are not treated with any drugs.

### Cell loading and confocal imaging

Devices were plasma treated prior to cell loading in order to make the channels hydrophilic and therefore easier to perfuse. Cells were then loaded by external pressure. After loading, cells that remained inside the reservoir were washed out. A continuous pool of media was added over the inlet and outlet wells to ensure no pressure differential existed in the channels. The spontaneous migration of cells was imaged under a confocal laser scanning microscope (Leica TCS SP8) using a 20x/0.75NA objective with a 1 μm z-step and 5 min interval for a total of 200 frames (16hrs36min). No external physical or chemical gradient was applied.

### Fluorescence profile projection and normalization

All images analyzed were taken with the same imaging parameters. The images were processed by custom macro and MATLAB scripts to obtain morphological and subcellular profiles. The 3D fluorescence images at each frame were projected through the z and y axes using a maximum projection method implemented in FIJI to generate the projection kymographs. We chose to use maximum projection as it captures the highest activity across the cell and eliminates the effect of volume distribution dynamics throughout the cell. The projected fluorescence distribution at each frame (each row of the kymograph) was plotted and normalized to 100 normalized length units and 1 normalized area unit under the profile curve. The profile is flipped to ensure the positive direction along the x-axis always corresponds to the direction of cell movement at each frame. The intentions of the normalization are 1) to prepare the profile in a way that shows the relative intensity across the dimension along with the cell movement, and 2) to eliminate the effect of the difference in cell length so that the profile is comparable across time and cells.

### Variables definition

Cell Speed: The cell speed at each frame (t_x_) is calculated by absolute distance that the cells travel in a 30 min interval (the cell location at t_x+15 min_ minus cell location at t_x-15 min_). Then all usable frames are averaged to get the average cell speed under different geometric conditions. At least 5 usable frames are needed to get the average speed for a certain geometric condition, otherwise the data will not be included.

Cell circularity and aspect ratio: The cell circularity and aspect ratio at each time frame are measured. Then all usable frames are averaged to get the average cell circularity and aspect ratio under different geometric conditions. At least 5 usable frames are needed to get the average circularity and aspect ratio for a certain geometric condition, otherwise the data will not be included.

Leading Half Area: The area under the leading half (x = 51 to x = 100) of the profile. As the total area under the profile is normalized to 1, a front area higher than 0.5 means the actin is relatively more intensified at the leading half than the trailing half of the cell. The leading half area at each time frame is measured first, then the leading half area of all usable frames are averaged to get the average leading half area of a cell.

Peak-to-peak Distance: The distance between the peak apices identified from the normalized profile. High peak-to-peak distance means a more polarized fluorescence profile with two peaks localized closer to the two ends (x = 0 and x = 100). Peak-to-peak distance equals to 0 when the profile only has one peak. The peak-to-peak distance at each time frame is measured first, then the peak-to-peak distance of all usable frames are averaged to get the average peak-to-peak distance of a cell.

Peak-to-peak Ratio: The ratio of the maximum normalized intensity of the peak at the leading edge over the maximum normalized intensity of the peak at the trailing edge. A higher peak-to-peak ratio means the cell has more intensified fluorescence peak over the leading edge compared to the trailing edge. The peak-to-peak ratio at each time frame is measured first, then the peak-to-peak ratio of all usable frames are averaged to get the average peak-to-peak ratio of a cell.

To determine whether a frame is usable, two criteria were applied: (1) the full cell has to be in the field of view for a complete 30 min interval centered around the frame, and (2) any dividing cells in the videos are noted and frames taken close to the division time point are not included.

### Statistical analysis

A one-way analysis of variance was performed on all multi-group box plots, and a Tukey–Kramer post-hoc analysis was performed to show significance between any two groups. A two sample t-test was performed on all two-group box plots. Pairwise chi-square tests were performed between the bar plots in Fig. 4C separately. *P* values < 0.05*/0.01**/0.001*** were considered statistically significant.

## Supplementary Information

Supplementary Information

## Data Availability

The datasets generated and analyzed during the current study are available from the corresponding author upon reasonable request.
