# Supplementary material for: Morphodynamic signatures of MDA-MB-231 single cells and cell doublets undergoing invasion in confined microenvironments
Source: Sci Rep. 2021 Mar 22;11:6529. doi: 10.1038/s41598-021-85640-5 (PMC7985374; doi:10.1038/s41598-021-85640-5)
Supplement: Supplementary file 1 — Supplementary Information [file 41598_2021_85640_MOESM1_ESM.docx]

**Morphodynamic Signatures of MDA-MB-231 Single Cells and Cell Doublets Undergoing Invasion in Confined Microenvironments**

Xingjian Zhang (xingjian.zhang@yale.edu), Trevor Chan (trevor.chan@yale.edu), and Michael Mak* ([michael.mak@yale.edu](mailto:michael.mak@yale.edu))

Department of Biomedical Engineering, Yale University, New Haven, CT


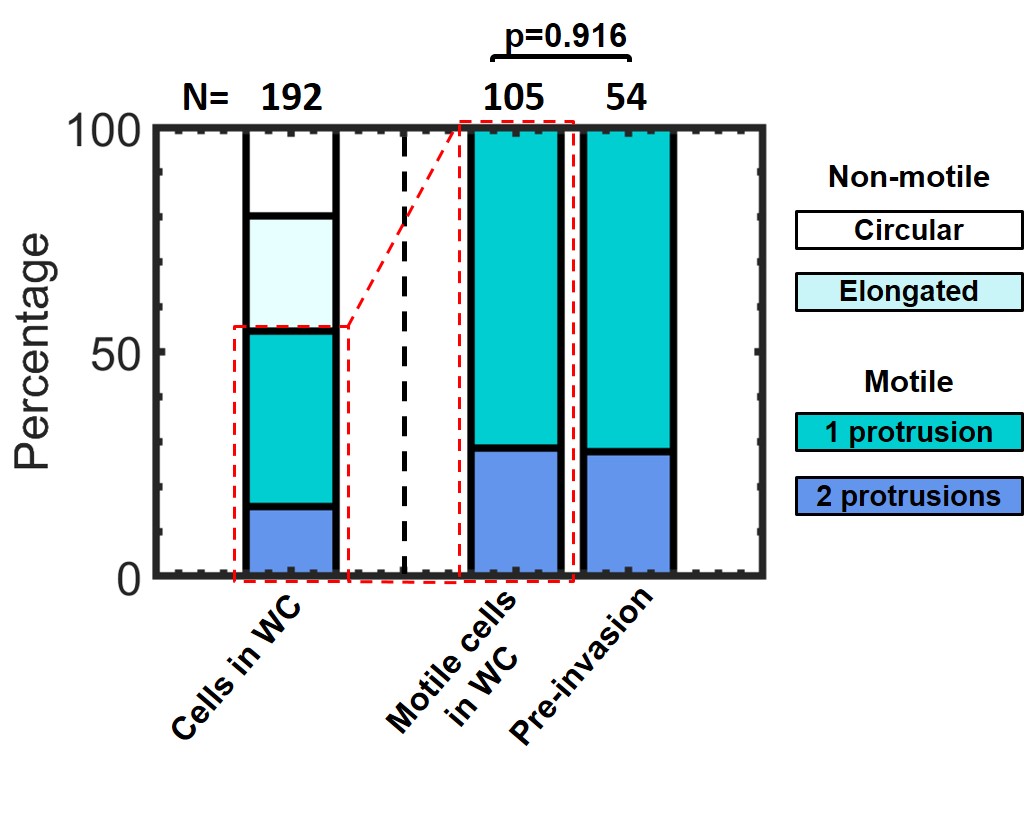


**Supplementary Figure 1. Cells in the wide channel vs. pre-invasion cells.** The cells in the wide channel (WC) group are cells that stay in the wide channel for at least 4hrs. Within the cell population, there are non-motile elongated or circular cells, with morphologies similar to those shown in Fig.1h, and there are motile cells. The motile subset of cells (with 1 or 2 leading protrusions) is used to compare with the cells in the pre-invasion stage in Fig. 4c. Cells in the pre-invasion stage are also motile as they migrate through the constrictions. The protrusion composition of cells in the pre-invasion stage is shown to distribute similarly in comparison to that of the motile subset of cells that stay inside the wide channel for more than 4hrs.
